# Incidence and treatment of group A streptococcal infections during covid-19 pandemic and 2022 outbreak: retrospective cohort study in England using OpenSAFELY-TPP

**DOI:** 10.1136/bmjmed-2023-000791

**Published:** 2024-05-24

**Authors:** Christine Cunningham, Louis Fisher, Christopher Wood, Victoria Speed, Andrew D Brown, Helen Curtis, Rose Higgins, Richard Croker, Ben FC Butler-Cole, David Evans, Peter Inglesby, Iain Dillingham, Sebastian CJ Bacon, Elizabeth Beech, Kieran Hand, Simon Davy, Tom Ward, George Hickman, Lucy Bridges, Thomas O'Dwyer, Steven Maude, Rebecca M Smith, Amir Mehrkar, Liam C Hart, Chris Bates, Jonathan Cockburn, John Parry, Frank Hester, Sam Harper, Ben Goldacre, Brian MacKenna

**Affiliations:** 1 Bennett Institute for Applied Data Science, Nuffield Department of Primary Care Health Sciences, University of Oxford, Oxford, UK; 2 NHS England, London, UK; 3 TPP, Leeds, UK

**Keywords:** COVID-19, Primary health care, Infectious disease medicine

## Abstract

**Objective:**

To investigate the effect of the covid-19 pandemic on the number of patients with group A streptococcal infections and related antibiotic prescriptions.

**Design:**

Retrospective cohort study in England using OpenSAFELY-TPP.

**Setting:**

Primary care practices in England that used TPP SystmOne software, 1 January 2018 to 31 March 2023, with the approval of NHS England.

**Participants:**

Patients registered at a TPP practice at the start of each month of the study period. Patients with missing data for sex or age were excluded, resulting in a population of 23 816 470 in January 2018, increasing to 25 541 940 by March 2023.

**Main outcome measures:**

Monthly counts and crude rates of patients with group A streptococcal infections (sore throat or tonsillitis, scarlet fever, and invasive group A streptococcal infections), and recommended firstline, alternative, and reserved antibiotic prescriptions linked with a group A streptococcal infection before (pre-April 2020), during, and after (post-April 2021) covid-19 restrictions. Maximum and minimum count and rate for each infectious season (time from September to August), as well as the rate ratio of the 2022-23 season compared with the last comparably high season (2017-18).

**Results:**

The number of patients with group A streptococcal infections, and antibiotic prescriptions linked to an indication of group A streptococcal infection, peaked in December 2022, higher than the peak in 2017-18. The rate ratios for monthly sore throat or tonsillitis (possible group A streptococcal throat infection), scarlet fever, and invasive group A streptococcal infection in 2022-23 relative to 2017-18 were 1.39 (95% confidence interval (CI) 1.38 to 1.40), 2.68 (2.59 to 2.77), and 4.37 (2.94 to 6.48), respectively. The rate ratio for prescriptions of first line, alternative, and reserved antibiotics to patients with group A streptococcal infections in 2022-23 relative to 2017-18 were 1.37 (95% CI 1.35 to 1.38), 2.30 (2.26 to 2.34), and 2.42 (2.24 to 2.61), respectively. For individual antibiotic prescriptions in 2022-23, azithromycin showed the greatest relative increase versus 2017-18, with a rate ratio of 7.37 (6.22 to 8.74). This finding followed a marked decrease in the recording of patients with group A streptococcal infections and associated prescriptions during the period of covid-19 restrictions where the maximum count and rates were lower than any minimum rates before the covid-19 pandemic.

**Conclusions:**

Recording of rates of scarlet fever, sore throat or tonsillitis, and invasive group A streptococcal infections, and associated antibiotic prescribing, peaked in December 2022. Primary care data can supplement existing infectious disease surveillance through linkages with relevant prescribing data and detailed analysis of clinical and demographic subgroups.

**WHAT IS ALREADY KNOWN ON THIS TOPIC**

- During the covid-19 pandemic, a substantial change occurred in the pattern of circulating viruses and bacteria that cause illnesses
- A peak in group A streptococcal infections in England starting in December 2022 was associated with 426 deaths, including 48 children, as of 7 May 2023
- Increased demand for antibiotics in this period resulted in medicines shortages and the introduction of serious shortage protocols
- Existing surveillance systems, such as notifiable disease reports and general practice in-hours surveillance bulletins, describe clinical events, but do not link to relevant prescribing data

**WHAT THIS STUDY ADDS**

- This study supports the findings of routine surveillance reports, indicating a reduction in group A streptococcal infections during covid-19 restrictions, followed by a peak in December 2022
- Antibiotic prescribing for an indication of group A streptococcal infection, particularly for phenoxymethylpenicillin alternatives and reserved antibiotics, was higher in the December 2022 peak than in the 2017-18 peak

**HOW THIS STUDY MIGHT AFFECT RESEARCH, PRACTICE, OR POLICY**

- The OpenSAFELY platform supported the response to an outbreak of infectious disease associated with the pandemic, giving detailed information on the recording of diseases and prescribing in general practice
- This framework can be reused or repurposed to provide near real time surveillance for future disease outbreaks or prescribing of any medication

## Introduction

During the covid-19 pandemic, a substantial change occurred in the pattern of circulating viruses and bacteria that cause illnesses.^1^ On 2 December 2022, the UK Health Security Agency (UKHSA) issued an alert of higher than normal events of scarlet fever and other group A streptococcal infections. On 5 December 2022, UKHSA issued an urgent public health message to health care professionals advising a lower threshold for prescribing antibiotics to children presenting with symptoms of group A streptococcal infection.^2^ Group A streptococcal infections are normally mild, often causing non-specific symptoms such as sore throat^3^ or, with scarlet fever, more specific symptoms (eg, strawberry tongue and a distinctive rash).^4^ On rare occasions, these bacteria can enter the bloodstream or deep tissues, causing invasive group A streptococcal infections, a serious infection that can cause death. Between 19 September 2022 and 7 May 2023, 426 deaths were associated with invasive group A streptococcal infections in England, including 48 children aged <18 years.^5^ UKHSA indicated that the increase likely reflected increased susceptibility to these infections in children because of the low numbers of group A streptococcal infections during the covid-19 pandemic, along with increased circulation of respiratory viruses.^1^ Before the covid-19 pandemic, 2017-18 was the last time substantially high numbers of group A streptococcal infections were reported,^5^ with a peak in notifications of scarlet fever in March 2018.^6^

Phenoxymethylpenicillin is recommended as the first line treatment for scarlet fever, with clarithromycin as an alternative in patients allergic to penicillin.^7^ After UKHSA issued their message in December 2022, increased demand leading to shortages of these drugs was anticipated. Interim treatment guidelines were produced by the NHS England Group A Streptococcal Clinical Reference Group and the UKHSA Incident Management Team,^8 9^ and a series of serious shortage protocols^10^ were introduced, both recommending a range of alternative antibiotics. The serious shortage protocols allowed community pharmacists to substitute an alternative antibiotic where the prescribed antibiotic was not available.

OpenSAFELY is a secure health analytics platform that allows near real time analysis of pseudonymised primary care patient records in England. We have previously shown that the OpenSAFELY platform can be used for rapid audit and feedback of national health incidents.^11^ We therefore used the OpenSAFELY platform to support the response to potential antibiotic shortages by rapidly investigating the effect of the covid-19 pandemic on the number of patients with group A streptococcal infections and related antibiotic prescriptions, recorded in primary care. This framework can be reused or repurposed for future outbreaks.

## Methods

### Study design

With the approval of NHS England, we performed a retrospective cohort study, in primary care practices in England, of group A streptococcal infections and prescribed antibiotics on a monthly basis, from 1 January 2018 to 31 March 2023. From 1 September 2022 to 31 March 2023, data were also extracted on a weekly basis to provide real time weekly results as part of the rapid surveillance report. Patients were included in a monthly or weekly data extract if they were alive and registered at a practice that used TPP SystmOne electronic health record software at the start of each week or month. Inclusion criteria were patients with recorded data for age and sex, and those aged ≤120 years. Data for sex were taken from information in the electronic health records rather than from patient reported gender.

### Data sources and processing

All data were linked, stored, and analysed securely within the OpenSAFELY platform (https://opensafely.org/).^12^ Data included pseudonymised data, such as coded diagnoses, medication use, and physiological parameters. No free text data were included. All code is shared openly for review and reuse under a Massachusetts Institute of Technology (MIT) open licence (https://github.com/opensafely/strepa_scarlet/). Detailed pseudonymised patient data are potentially re-identifiable and therefore are not shared. Aggregated data used in the analyses are available on reasonable request to the authors. Data management and analysis was performed with Python 3.

### Identification of clinical events

Based on the recommendation of NHS England, UKHSA interim clinical guidance,^8 9^ and the phenoxymethylpenicillin serious shortage protocols,^10^ we generated dictionary of medicines and devices (dm+d) codelists for the oral formulations of these antibiotics: phenoxymethylpenicillin, amoxicillin, clarithromycin, erythromycin, azithromycin, flucloxacillin, cefalexin, and co-amoxiclav. Antibiotics were also categorised into three groups: phenoxymethylpenicillin (group 1) as the first line antibiotic for group A streptococcal infections; macrolides, amoxicillin, and flucloxacillin (group 2) as the antibiotics recommended if group 1 antibiotics were not available or if patients were allergic to penicillin; and cefalexin and co-amoxiclav (group 3) as the least preferred broad spectrum antibiotics because of their greater risk of promoting antimicrobial resistance.^8^

We also developed three SNOMED CT (Systematised Medical Nomenclature for Medicine Clinical Terms) codelists to identify the different types of group A streptococcal infections or possible infections: sore throat or tonsillitis (possible group A streptococcal throat infection); scarlet fever; and invasive group A streptococcal infections. In the absence of a clear indicator of a group A streptococcal throat infection, we took a pragmatic approach, and codes for sore throat, pharyngitis, or tonsillitis were included when an alternative cause for the sore throat was not stated (eg, staphylococcal tonsillitis), but non-group A streptococcal infections could have been included. Scarlet fever and invasive group A streptococcal infections have clearer diagnosis codes, but because the invasive type of infection is more likely to occur in hospital than in primary care, some patients might be missing from this analysis. All three codelists were included to provide a sensitive measure that would more likely provide an early indicator of changes in the number of patients with group A streptococcal infections.

Online supplemental table S1 shows the medication and clinical codelists used in this analysis. The codelists are openly available for review and reuse (https://www.opencodelists.org/). Prescribing data were based on prescriptions issued within the electronic health record, and do not necessarily reflect whether the prescription was dispensed. Clinical events data were based on clinical codes added to a patient's record. These codes are often added by a clinician during a consultation to indicate the presence of a sign or symptom (eg, sore throat), or that a clinical diagnosis has been made (eg, scarlet fever). These codes do not necessarily indicate positive test results.

10.1136/bmjmed-2023-000791.supp1 Supplementary data

### Outcomes

#### Clinical events

For each time period, we identified patients with clinical codes for each of the events, sore throat or tonsillitis, scarlet fever, or invasive group A streptococcal infection. A separate measure was computed of patients with any of the above codes, which we defined as a patient with group A streptococcal infection; if a patient had tonsillitis and scarlet fever coded as two separate events in the same month, they were only counted once for this outcome. To analyse the rate of antibiotic prescriptions, we also counted the number of patients with each clinical event (scarlet fever, sore throat or tonsillitis, or invasive group A streptococcal infection) who also had an antibiotic prescription up to seven days before or 14 days after the clinical event.

#### Antibiotics

For each time period, we identified patients who had a record of individual antibiotics and we also counted patients with any antibiotic. We then grouped the antibiotics into group 1 (first line), group 2 (alternative), and group 3 (reserved broad spectrum). Although all of the antibiotics in this study can be used to treat group A streptococcal infections, this use is not exclusive. Therefore, the results focus on antibiotic prescribing in combination with any of the clinical events of interest up to 14 days before or seven days after the prescribing event.

#### Analysis by demographic subgroup

We extracted the following patient demographic information: age (0-4, 5-9, 10-14, 15-44, 45-64, 65-74, and ≥75 years), sex, 2019 index of multiple deprivation based on the patient's address (categorised as groups 1-5, with group 1 being the most deprived), ethnic group according to the 2001 census categories (white, mixed, Asian or Asian British, black or black British, Chinese or other, or unknown), and the region of the patient's practice, based on the nine Nomenclature of Territorial Units for Statistics regions for England (North East, North West, Yorkshire and the Humber, East Midlands, West Midlands, East, London, South East, and South West). Demographic subgroup breakdowns for all outcomes are available in supporting dashboards (https://reports.opensafely.org/reports/scarlet-fever-and-invasive-group-a-strep-cases-throughout-the-covid-19-pandemic/, https://reports.opensafely.org/reports/scarlet-fever-and-invasive-group-a-strep-cases-throughout-the-covid-19-pandemic-weekly/). In this paper, we present prescribing information for phenoxymethylpenicillin for an indication of group A streptococcal infection, grouped by age, region, ethnic group, and index of multiple deprivation.

Patients with missing values in each demographic group were excluded from the plots, but are described in table 1. The OpenSAFELY-TPP population has been shown to be broadly representative of the English population, but some differences in regional coverage exist based on the use of the electronic health record system, with the highest coverage in the East of England (91%) and lower coverage in London (19%).^13^ Care should be taken when interpreting regional rates because they could reflect differences in other personal characteristics.

**Table 1:** Characteristics of registered patients and rates of group A streptococcal infections (ie, sore throat or tonsillitis, scarlet fever, or invasive streptococcal infection) at the end of the study period (31 March 2023)

	No (%) of registered patients in overall population	Rate per 1000 (95% CI) patients with group A streptococcal infections
Total No of patients	25 541 940 (100.0)	3.36 (3.34 to 3.38)
Sex		
Women	12 748 390 (49.9)	4.09 (4.06 to 4.13)
Men	12 793 540 (50.1)	2.63 (2.60 to 2.65)
Region		
East	5 857 920 (22.9)	3.73 (3.68 to 3.78)
East Midlands	4 447 350 (17.4)	3.36 (3.31 to 3.42)
London	1 819 560 (7.1)	2.60 (2.53 to 2.67)
North East	1 189 140 (4.7)	3.43 (3.33 to 3.54)
North West	2 209 000 (8.6)	3.92 (3.84 to 4.01)
South East	1 656 800 (6.5)	2.88 (2.80 to 2.96)
South West	3 532 570 (13.8)	2.59 (2.54 to 2.65)
West Midlands	1 035 480 (4.1)	3.85 (3.73 to 3.97)
Yorkshire and the Humber	3 705 650 (14.5)	3.58 (3.52 to 3.64)
Missing	88 460 (0.3)	3.73 (3.33 to 4.13)
Index of multiple deprivation		
1 (most deprived)	5 044 510 (19.7)	4.05 (4.00 to 4.11)
2	4 928 760 (19.3)	3.45 (3.40 to 3.50)
3	5 231 200 (20.5)	3.12 (3.07 to 3.17)
4	4 909 180 (19.2)	2.97 (2.92 to 3.02)
5 (least deprived)	4 516 700 (17.7)	2.81 (2.76 to 2.86)
Missing	911 590 (3.6)	5.21 (5.06 to 5.36)
Ethnic group		
Black	708 560 (2.8)	3.02 (2.89 to 3.15)
Mixed	492 010 (1.9)	5.00 (4.80 to 5.20)
Other	621 040 (2.4)	3.01 (2.87 to 3.15)
South Asian	2 064 330 (8.1)	4.31 (4.22 to 4.40)
White	19 669 920 (77.0)	3.38 (3.35 to 3.40)
Missing	1 986 080 (7.8)	2.03 (1.97 to 2.10)
Age (years)		
0-4	1 199 240 (4.7)	11.85 (11.66 to 12.04)
5-9	1 433 440 (5.6)	11.07 (10.90 to 11.24)
10-14	1 537 990 (6.0)	5.68 (5.56 to 5.80)
15-44	9 996 000 (39.1)	3.64 (3.60 to 3.67)
45-64	6 549 540 (25.6)	1.18 (1.15 to 1.21)
65-74	2 465 340 (9.7)	0.73 (0.69 to 0.76)
≥75	2 360 370 (9.2)	0.47 (0.44 to 0.49)

Patients with more than one of the same clinical event in a time period were only counted once.

Counts were rounded to the nearest 10 and the rate computed with the rounded numbers.

Sums of category counts might not exactly match the total because of rounding.

CI, confidence interval.

#### Statistical methods

For each time period (month or week), the number of infections and prescriptions were described with simple descriptive statistics of counts and crude rates. Counts represent the number of patients with at least one prescription or clinical event of interest in a specific time period. Counts ≤5 were redacted and all numbers rounded to the nearest 10 to avoid potential re-identification of patients. To compute the crude rate for each time period, the rounded count was divided by the included study population and multiplied by 1000 to achieve a rate per 1000 registered patients. All rates were calculated with 95% confidence intervals (CIs). Rates were used to account for the registered OpenSAFELY-TPP population increasing over time (online supplemental figure S1).

We computed the maximum and minimum count and rate for each infectious season (time from September to August),^5^ and compared the years before the covid-19 restrictions with the covid-19 restriction years and the years after the covid-19 restrictions. The covid-19 restriction period was defined as 1 April 2020 to 1 April 2021, when non-essential services began reopening.^14^ The rate ratio of the 2022-23 season maximum, to the maximum rate of the last comparably high season (2017-18) was also calculated.

#### Dashboard

Regular dashboards have been produced and were routinely updated at https://reports.opensafely.org/ with near real time data from January to April 2023. These dashboards have separate sections for each type of group A streptococcal infection and antibiotic, as well as additional graphs. An example of an additional plot is top codes over time. Each codelist is composed of a number of codes. Scarlet fever, for example, can be coded in the patient's medical record with a SNOMED CT code (eg, 1087781000000109 for suspected scarlet fever, 170523002 for notification of scarlet fever). The number of times each code was used was summed across the whole study period, and the count and proportion of the top five most commonly used codes were reported. The top five codes in the first month and the top five codes in the last month were then plotted over the whole study period.

### Patient and public involvement

OpenSAFELY has involved patients and the public in various ways: we developed a public website that provides a detailed description of the platform in language suitable for a lay audience (https://opensafely.org); we have participated in two citizen juries exploring public trust in OpenSAFELY; we have co-developed an explainer video (https://www.opensafely.org/about/); we have patient representation who are experts by experience on our OpenSAFELY Oversight Board; we have partnered with Understanding Patient Data to produce lay explainers on the importance of large datasets for research; we have presented at various online public engagement events to key communities (eg, Healthcare Excellence Through Technology; Faculty of Clinical Informatics annual conference; NHS Assembly; HDRUK symposium); and more. To ensure the patient voice is represented, we are working closely to decide on language choices with appropriate medical research charities (eg, Association of Medical Research Charities). We will share information and interpretation of our findings through press releases, social media channels, and plain language summaries.

## Results

At the start of the study period, 23 816 470 patients were alive and registered in OpenSAFELY-TPP with no missing data for age or sex. This number increased to 25 541 940 at the end of the study period on 31 March 2023 (table 1), representing about 40% of the total number of patients registered at a general practice in England.^15 16^ In March 2023, 3650 patients had a record of scarlet fever, 83 080 sore throat or tonsillitis, and 70 had an invasive group A streptococcal infection, affecting 86 800 (3.4 per 1000) patients in total. Clinical codes for these infections were most common among ages 0-4 years (11.8 per 1000) and 5-9 years (11.1 per 1000). Women had a higher rate than men (4.1 per 1000 *v* 2.6 per 1000), and clinical events increased with deprivation, ranging from 2.8 per 1000 in the least deprived group to 4.1 per 1000 in the most deprived group. The South West, London, and the South East regions had the lowest crude rates of clinical events (2.6, 2.6, and 2.9 per 1000, respectively). The highest rate of clinical events was in those from the mixed ethnic group (5.0 per 1000), followed by South Asian (4.3 per 1000), white (3.4 per 1000), black (3.0 per 1000), and other (3.0 per 1000) groups.

### Trends in clinical events

**Figure 1:**
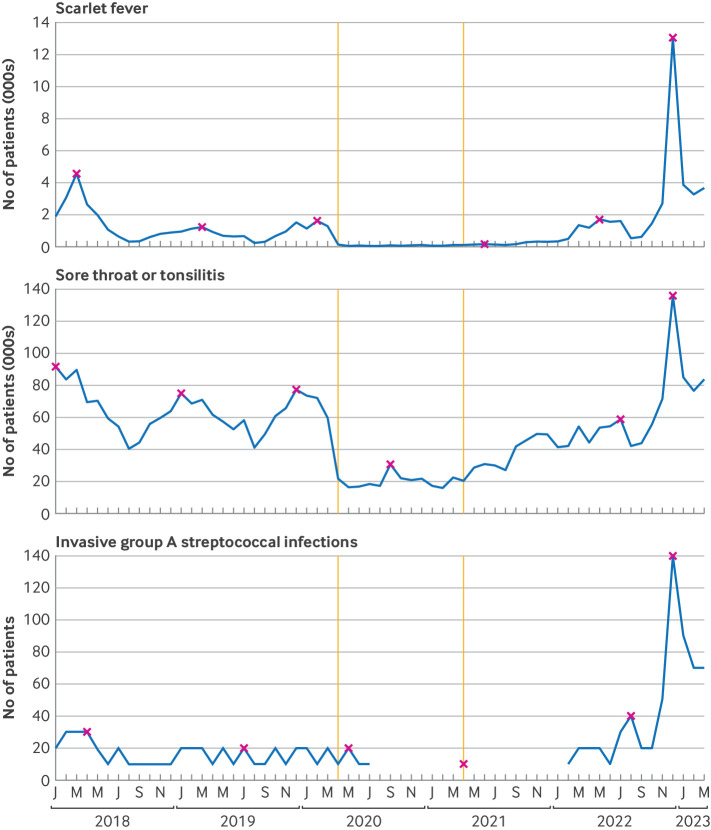
Monthly numbers of patients with scarlet fever, sore throat or tonsillitis, or invasive group A streptococcal infection. Vertical lines represent the start of the covid-19 restriction (April 2020) and recovery (April 2021) periods. Maximum value of each season (September to August) is indicated (x). Gaps indicate data were redacted because of low counts

In the three infectious seasons (2017-18, 2018-19, and 2019-20) before covid-19 restrictions, the number of patients with sore throat or tonsillitis for each month peaked each winter, at 91 510 (January 2018), 75 070 (January 2019), and 77 470 (December 2019) (figure 1). In 2020-21, which included the covid-19 restriction period (April 2020 to March 2021), the typical winter peak was not seen, with a maximum count of only 30 860 in September 2020, lower than the minimum in the previous seasons (eg, 40130 in August 2018 and 41 000 in August 2019). The count increased in 2021-22 but with no distinct seasonal pattern. Starting in September 2022, we found a steep increase in events, peaking in December 2022 at 135 860, 44 350 higher than the previous highest count (January 2018), a rate ratio of 1.39 (95% CI 1.38 to 1.40) (online supplemental table S2). We also found that 75% of these patients with sore throat or tonsillitis also had an antibiotic prescription up to seven days before or 14 days after the clinical event (online supplemental table S3), but these values might include patients with non-group A streptococcal infections. This percentage remained largely consistent between January 2018 and March 2023, but showed a small increase in the winter 2022-23 season (online supplemental figure S2a).

Scarlet fever events similarly followed a seasonal pattern that was not seen during the covid-19 restriction period. The maximum number of patients in the 2020-21 season was 150 in June 2021, lower than the minimum of 260 in August 2019 before the covid-19 pandemic (online supplemental table S2). The number of events then peaked in December 2022 at 13 040, a rate ratio of 2.68 (95% CI 2.59 to 2.77) compared with a peak of 4570 before the covid-19 pandemic in March 2018. We found that 90% of the scarlet fever codes also had an antibiotic prescription up to seven days before or 14 days after the clinical event (online supplemental table S3). This percentage remained consistent between January 2018 and March 2023 (online supplemental figure S2a)

Recorded invasive group A streptococcal infections peaked at 140 in December 2022, a rate ratio of 4.37 (95% CI 2.94 to 6.48) compared with the 2017-18 peak of 30 (online supplemental table S2). Only 29% of codes for invasive group A streptococcal infection had an antibiotic prescription seven days before or 14 days after the clinical event in the primary care record (online supplemental table S3).

Overall, recording of codes for any indication of group A streptococcal infection in primary care peaked in December 2022 at 146 260 (5.7 per 1000). This value represented a 1.5-fold increase from the last comparably high season in 2017-18 (93 340 or 3.9 per 1000) (online supplemental table S2).

### Antibiotics used to treat group A streptococcal infections

**Figure 2:**
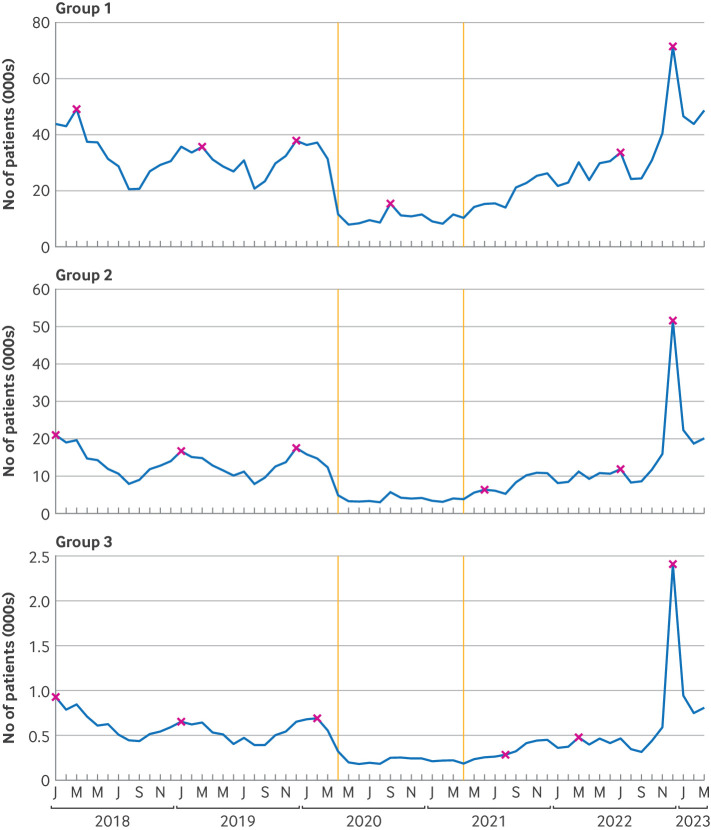
Monthly numbers of patients with an antibiotic prescription and a record of scarlet fever, sore throat or tonsillitis, or invasive group A streptococcal infection, up to 14 days before or seven days after the prescribing event. Antibiotics were categorised into three groups: phenoxymethylpenicillin (group 1), the first line antibiotic for group A streptococcal infection; macrolides, amoxicillin, and flucloxacillin (group 2), antibiotics recommended if group 1 antibiotics were not available or if patients were allergic to penicillin; and cefalexin and co-amoxiclav (group 3), reserved broad spectrum antibiotics. Vertical lines represent the start of the covid-19 restriction (April 2020) and recovery (April 2021) periods. Maximum values of each season (September to August) are indicated (x)

Figure 2 shows counts of antibiotics prescribed with a clinical indication of group A streptococcal infection by antibiotic group. The percentage of all prescriptions that had an indication for a group A streptococcal infection varied by antibiotic; phenoxymethylpenicillin (group 1) had the highest in March 2023 (41%) and flucloxacillin (group 2) had the lowest (<1%, online supplemental table S3 and online supplemental figure S2b). The same plot of antibiotics prescribed with or without a relevant indication for group A streptococcal infection (online supplemental figure S3) and other metrics are available in the dashboard (https://reports.opensafely.org/reports/scarlet-fever-and-invasive-group-a-strep-cases-throughout-the-covid-19-pandemic/) and in the online supplemental material for context.

Before covid-19 restrictions, the highest level of group 1 prescribing with a clinical indication of group A streptococcal infection was 48 990 patients in March 2018 followed by seasonal highs of 35 680 patients in January 2019 and 37 910 in December 2019. The 2020-21 season peak of 15 610 in September 2020 was lower than any minimum count before the covid-19 pandemic. Counts increased over 2021-22 but with no distinct seasonal pattern. The peak of 71 440 patients in December 2022 compared with the 2017-18 peak had a rate ratio of 1.37 (95% CI 1.35 to 1.38) (online supplemental table S4). In December 2022, prescribing of group 2 antibiotics with an indication of group A streptococcal infection (51 690) was 2.30 (2.26 to 2.34) times the previous high (21 000 in January 2018), and prescribing of group 3 antibiotics (2410) was 2.42 (2.24 to 2.61) times the previous high (930 in January of 2018).

Comparing the 2017-18 peak with the 2022 peak for individual antibiotics with an indication of streptococcal infection (online supplemental table S5), azithromycin had the largest increase in rate (rate ratio 7.37, 95% CI 6.22 to 8.74), followed by cefalexin (3.81, 3.33 to 4.35), amoxicillin (2.53, 2.47 to 2.59), clarithromycin (2.16, 2.10 to 2.22), co-amoxiclav (1.85, 1.69 to 2.04), erythromycin (1.71, 1.65 to 1.78), flucloxacillin (1.49, 1.30 to 1.70), and phenoxymethylpenicillin (1.37, 1.35 to 1.38).

### Analysis by personal characteristicsdemographic subgroups

**Figure 3:**
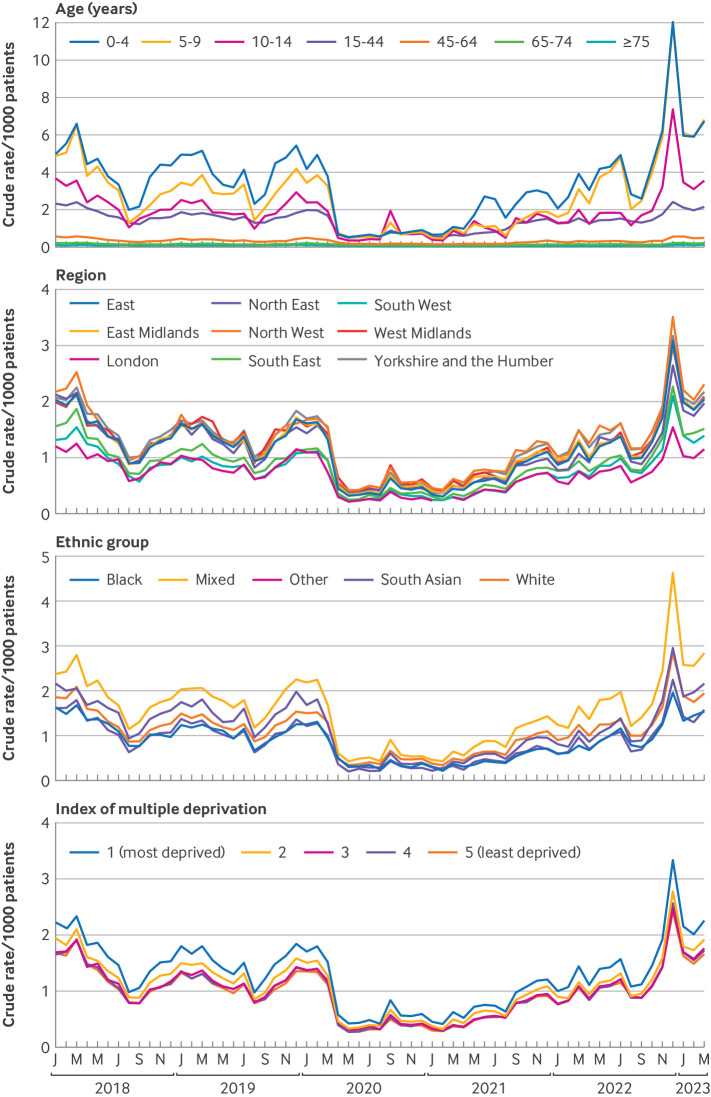
Monthly rate per 1000 patients with a recorded prescription of phenoxymethylpenicillin and a record of scarlet fever, sore throat or tonsillitis, or invasive group A streptococcal infection, up to 14 days before or seven days after the prescribing event, by age, region, ethnic group, and index of multiple deprivation

The crude rate of phenoxymethylpenicillin prescribed for an indication of group A streptococcal infection over time (figure 3) increased with increasing deprivation and was lowest in London, the South West, and the South East. The rate was highest in those from the mixed ethnic group, followed by South Asian, white, black, and other groups. These patterns were similar throughout the study period (online supplemental table S6).

The rate increased with decreasing age, with children aged 0-4 years showing the highest rate throughout the study period. During the 2018-19 and 2019-20 seasons, the rate of prescribing of phenoxymethylpenicillin was higher in those aged 0-4 years than 5-9 years. During the high seasons (2017-18 and 2022-23), prescribing for an indication of group A streptococcal infection was similar in those aged 0-4 years and 5-9 years (online supplemental table S7).

### Dashboard

**Figure 4:**
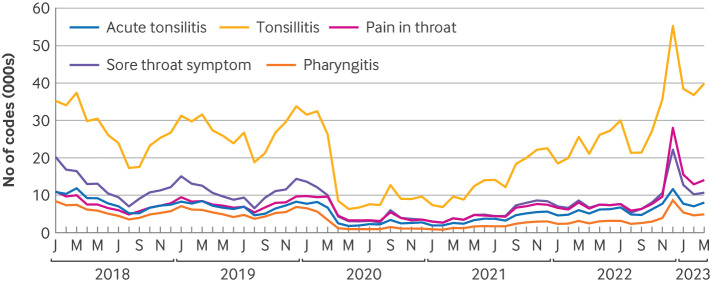
Monthly number of codes for sore throat and tonsillitis

Our regularly updated dashboards have more detailed analysis of each type of group A streptococcal clinical event and associated antibiotics (https://reports.opensafely.org/reports/scarlet-fever-and-invasive-group-a-strep-cases-throughout-the-covid-19-pandemic/ and https://reports.opensafely.org/reports/scarlet-fever-and-invasive-group-a-strep-cases-throughout-the-covid-19-pandemic-weekly/). Figure 4 is an example of a more detailed graph, showing trends over time for the most commonly used sore throat and tonsillitis codes. Tonsillitis was the most commonly recorded code over the study period. The individual code trends generally followed the total trend, peaking in winter and dropping during the covid-19 restriction period. Before covid-19 restrictions, 12% of total codes were for pain in the throat, which increased sharply to 20% in April 2020, driven by a decrease in the monthly number of recorded codes for tonsillitis from around 27 000 to around 9000. The proportion gradually decreased to 13% during covid-19 restrictions, before peaking again to 20% in December 2022. These dashboards were used by NHS England to monitor real time demand for antibiotics and to estimate the relative increase in demand above the expected seasonal variation. The data were used to inform mathematical modelling of demand and forecasting of supply chain resilience, in collaboration with the pharmaceutical industry, to guide strategic decisions about the design and implementation of serious shortage protocols.

## Discussion

### Principal findings

Recording of codes for an indication of group A streptococcal infection in primary care peaked in December 2022 at 146 260 (5.7 per 1000). This value represented a 1.5-fold increase from the last comparably high season in 2017-18 (93 340 or 3.9 per 1000). Before the covid-19 pandemic, group A streptococcal infections followed a seasonal pattern, peaking sometime between December and March. During the period of covid-19 restrictions, we saw a marked decrease in the recording of patients with group A streptococcal infections and treatments, with the maximum counts and rates lower than any minimum values recorded before the covid-19 pandemic, and no distinct seasonal pattern.

Prescriptions of first line antibiotics in patients with an indication of group A streptococcal infection peaked at 2.80 per 1000 (rate ratio 1.37, 95% CI 1.35 to 1.38), alternative antibiotics at 2.03 per 1000 (2.30, 2.26 to 2.34), and reserved antibiotics at 0.09 per 1000 (2.42, 2.24 to 2.61). For individual antibiotics, azithromycin with an indication of group A streptococcal infection showed the greatest relative increase (rate ratio 7.37, 6.22 to 8.74). This finding followed a marked decrease in the recording of patients with group A streptococcal infections and associated prescriptions during the period of covid-19 restrictions when the maximum count and rates were lower than any minimum rates before the covid-19 pandemic.

### Findings in context

Scarlet fever and invasive group A streptococcal infection are notifiable diseases in England, meaning that registered practitioners have a statutory duty to report suspected cases to the local council or health protection team by submitting a notification form or by telephone.^17^ UKHSA publishes weekly and summary notifications of infectious diseases reports for England and Wales, with detailed analysis by region, county, and local authority. From 12 September to 4 December 2022, 659 notifications of invasive group A streptococcal infections in England (1.2 per 100 000) and 6601 (11.7 per 100 000) notifications of scarlet fever were recorded.^6^ In OpenSAFELY-TPP from 1 September to 30 November 2022, 90 patients with invasive group A streptococcal infections (average rate 0.4 per 100 000) and 4750 patients with scarlet fever (average rate 15 per 100 000) were recorded. The lower rate of invasive group A streptococcal infections in the primary care data is expected because patients would normally be managed in secondary care. The higher rate of recordings of scarlet fever is also unsurprising, because disease notifications might be delayed or suspected infections might not be reported. The higher rate of patients with scarlet fever in our report highlights the value of primary care data for real time monitoring of disease. By including all codelists for sore throat or tonsillitis, scarlet fever, and invasive group A streptococcal infections to improve sensitivity, an early indicator of changes in patient numbers might be identified for decision makers. Further research on how notifications of infectious disease report compare with data in the primary care record is needed.

Daily incidence rates of group A streptococcal infections are reported in general practice in-hours syndromic surveillance bulletins,^18^ weekly reports published by UKHSA with a sample of about 650 practices covering seven million registered patients in England. The reports include analysis by age and UKHSA region, and similar to our OpenSAFELY data, diagnoses might not be laboratory confirmed. Our overall findings for scarlet fever were comparable. Our combined clinical indicator (scarlet fever, sore throat or tonsillitis, and invasive group A streptococcal infections) showed a higher rate because of inclusion of a broader range of clinical codes and conditions than the most similar indicators in the UKHSA report. Our OpenSAFELY-TPP report complements UKHSA reports by: reporting on a larger sample of practices; including tonsillitis in our sore throat codelist (figure 4), which might decrease our specificity, but increased our sensitivity; linking antibiotic prescribing codes to diagnosis codes at the patient level, allowing us to monitor antibiotic prescribing related to group A streptococcal infections; providing more detailed analysis of data (whereas the general practice in-hours report provides a quick summary for general practitioners of which syndromes might be above or below baseline); and sharing all of our analytic code and codelists openly for examination, comparison, and reuse (online supplemental table S1).

Lower levels of circulating viruses during covid-19 restrictions might have contributed to a peak in patients with group A streptococcal infections in December 2022. Although two years have passed since restrictions began to ease in England, the ongoing monitoring of infectious diseases and the prescription of associated treatments will continue to be important, although non-seasonal patterns of viruses are still seen.

### Strengths and weaknesses of this study

The main strength of this study was the speed and scale of its delivery to the NHS outbreak team. The time from project approval to first report was seven days. This study was implemented across the full electronic health record coded data covering 40% of general practices in England. The report was, and can in future be, delivered on a weekly basis, providing a near real time warning system for future outbreaks and pressures on the supply of antibiotics. Because this dashboard was developed for a small outbreak team, user research into how clinicians and decision makers could best make use of this report or similar surveillance reports could improve future versions.

In the absence of a clear indicator of group A streptococcal throat infection, a codelist for sore throat or tonsillitis was developed when an alternative cause was not stated (eg, staphylococcal tonsillitis). Because these symptoms can often be caused by viruses, including covid-19, we used the clinical codelists in combination with codelists for antibiotics recommended for treatment of group A streptococcal infections to improve specificity. In March 2023, 75% of these codes were associated with an antibiotic prescription, but infections might still be viral and not bacterial. Our codelists probably captured some non-group A streptococcal events, which is why we reported the total number of patients with infections as well as the clinical events divided into invasive group A streptococcal infections, scarlet fever, and sore throat or tonsillitis. Also, our approach relied on a clinician adding an appropriate clinical code to indicate a diagnosis, but coding of consultations varies, and some consultations might not be coded.

Our study looked at antibiotic prescriptions issued, but prescriptions recorded in the primary care record might not always be dispensed, or in some cases the dispensed item might differ from the prescribed item because of the use of a serious shortage protocol.^10^ Serious shortage protocols for phenoxymethylpenicillin were in place in the UK from 15 December 2022 to 12 May 2023, allowing for other formulations of phenoxymethylpenicillin, or substitution with amoxicillin, flucloxacillin, cefalexin, co-amoxiclav, erythromycin and, up until mid-January, azithromycin and clarithromycin. In two recent freedom of information requests,^19 20^ we found that from December 2022 to March 2023, 35 458 items were dispensed under these serious shortage protocols across England, 23 583 of which were not a phenoxymethylpenicillin formulation (online supplemental table S8 and https://github.com/opensafely/strepa_scarlet/tree/main/analysis/ssp-analysis). In the same months in OpenSAFELY-TPP (40% of general practices in England), more than 530 000 prescriptions of phenoxymethylpenicillin were recorded. If we assume that prescribing reported in OpenSAFELY-TPP is representative of overall prescribing, then the substitution of phenoxymethylpenicillin with a non-phenoxymethylpenicillin alternative would represent less than 2% of phenoxymethylpenicillin prescriptions. Therefore, although this analysis was based on prescriptions and not dispensings, serious shortage protocols likely had minimal effects on the findings. Currently, no national data linking prescriptions to dispensed items exist^21^; we encourage collection of these data to understand the effect of serious shortage protocols in future research.

In this study, we did not identify infections by diagnostic tests, test results, or clinical scoring tools. Throat swabs are recommended where the diagnosis is uncertain,^8^ but a diagnosis of scarlet fever and other group A streptococcal infections is based on clinical criteria. Clinical scoring tools, such as feverPAIN, are recommended for determining the likelihood that antibiotics would help a patient, and during the December 2022 outbreak, the feverPAIN prescribing threshold was lowered from four to three.^8^ Future research could investigate the availability and usefulness of relevant diagnostic tests and clinical scoring tools in electronic health records, and whether they can help improve the specificity of our analyses. Future work could also make use of other data sources, such as onward presentations at hospitals.

### Policy implications

OpenSAFELY is a secure health analytics platform that allows near real time analysis of pseudonymised primary care patient records in England to support the response to the covid-19 pandemic. We have previously shown that the OpenSAFELY platform can be used for rapid audit and feedback.^11^ Here, we have shown that OpenSAFELY can support the response to an outbreak of infectious disease associated with the pandemic, giving detailed information on disease recording and prescribing in general practice. Information about demand for liquid formulations of antibiotics influenced the design of the serious shortage protocols and prompted the development of clinical guidance by the NHS Specialist Pharmacy Service on administration of solid dose forms to children. This software framework can be reused or repurposed to provide near real time surveillance for future disease outbreaks or prescribing of any medications.

### Conclusions

We found that before the covid-19 pandemic, sore throat or tonsillitis, scarlet fever, and invasive group A streptococcal infections followed a seasonal pattern, peaking sometime between December and March. During the period of covid-19 restrictions, a marked decrease in infections was seen, with the maximum counts and rates lower than any minimum rates before the covid-19 pandemic. Patients with group A streptococcal infections increased in 2021-22 but with no distinct seasonal pattern, until peaking in December 2022. Primary care data can supplement existing infectious disease surveillance reports by linking with patient level prescribing data. We produced a live updating dashboard with more detailed analysis and sensitive codelists to provide greater context to relevant analysts and outbreak teams.

## Data Availability

No data are available. Access to the underlying identifiable and potentially re-identifiable pseudonymised electronic health record data is tightly governed by various legislative and regulatory frameworks, and restricted by best practice. The data in the NHS England OpenSAFELY COVID-19 service are from general practice data across England where TPP is the data processor.
