# Supplementary material for: Incidence and treatment of group A streptococcal infections during covid-19 pandemic and 2022 outbreak: retrospective cohort study in England using OpenSAFELY-TPP
Source: BMJ Med. 2024 May 24;3(1):e000791. doi: 10.1136/bmjmed-2023-000791 (PMC11129040; doi:10.1136/bmjmed-2023-000791)
Supplement: Supplementary data [file bmjmed-2023-000791supp001.pdf]

Supplemental

Figure S1. Population denominator in OpenSAFELY-TPP from January 2018 through March 2023. Patients who have died, or have missing age or sex have been excluded

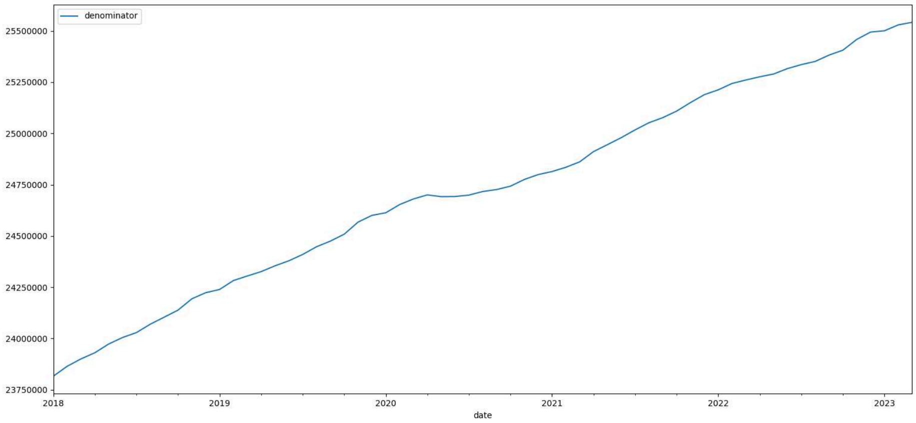

Figure S2. (a) Percentage of prescriptions that had a GAS indication by antibiotic between January 2018 and March 2023. (b) Percentage of GAS clinical events prescribed an antibiotic between January 2018 and March 2023.

(a)

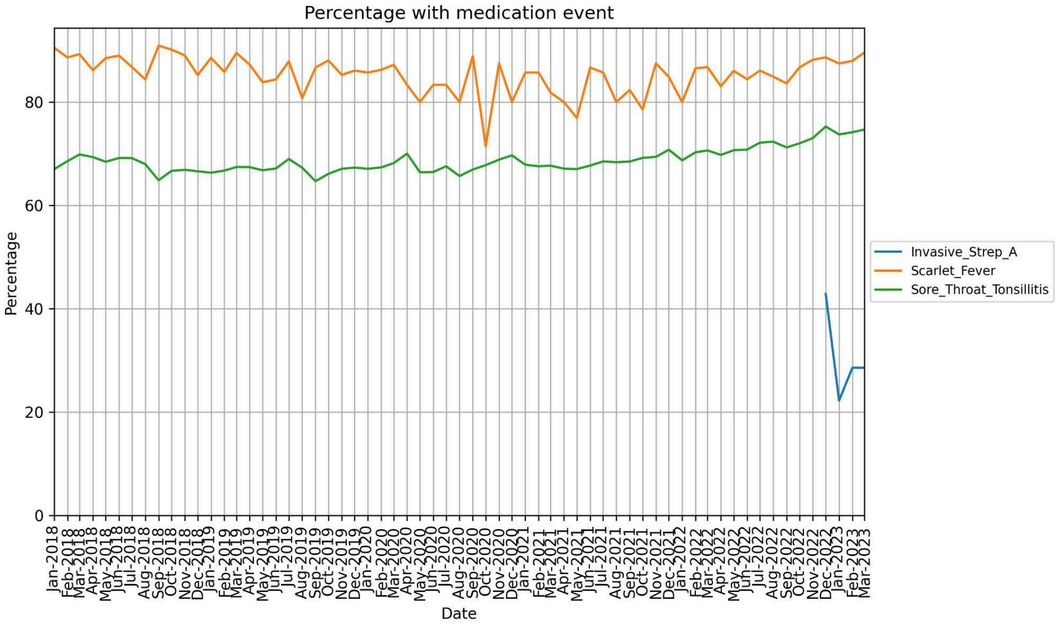

(b)

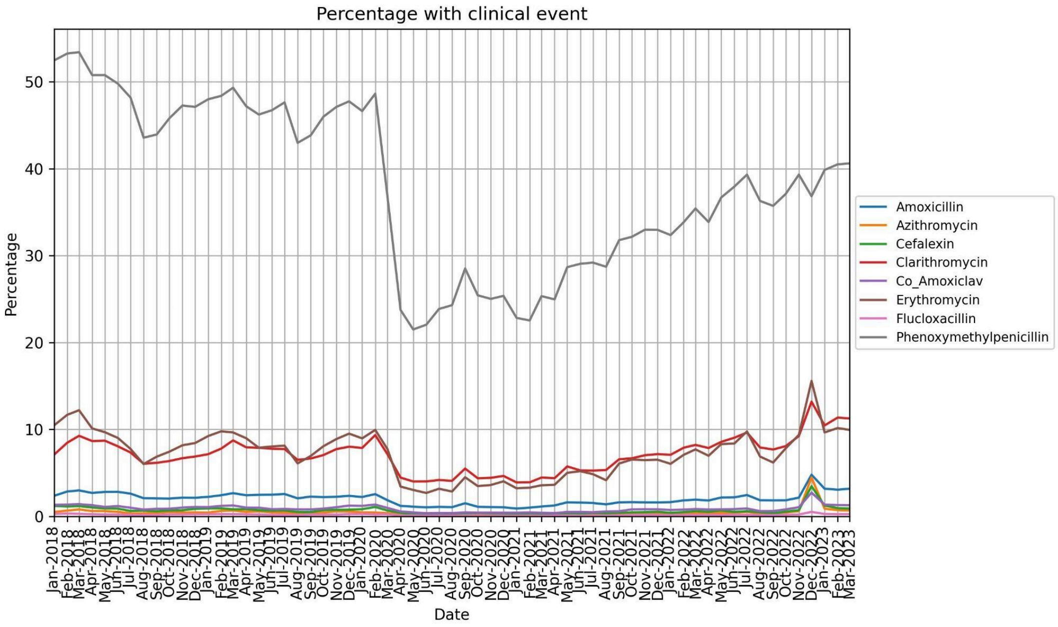
Percentage with clinical event
Percentage
0
10
20
30
40
50
Date
Amoxicillin
Azithromycin
Cefalexin
Clarithromycin
Co_Amoxiclav
Erythromycin
Flucloxacillin
Phenoxymethylpenicillin

Figure S3: Monthly count of patients with an antibiotic prescription (with or without GAS indication). Antibiotics have been grouped into Group 1, Group 2, and Group 3. Vertical lines represent the start of the COVID-19 restriction period (April 2020) and recovery period (April 2021). The maximum value of each season (September through August) is annotated with an x.

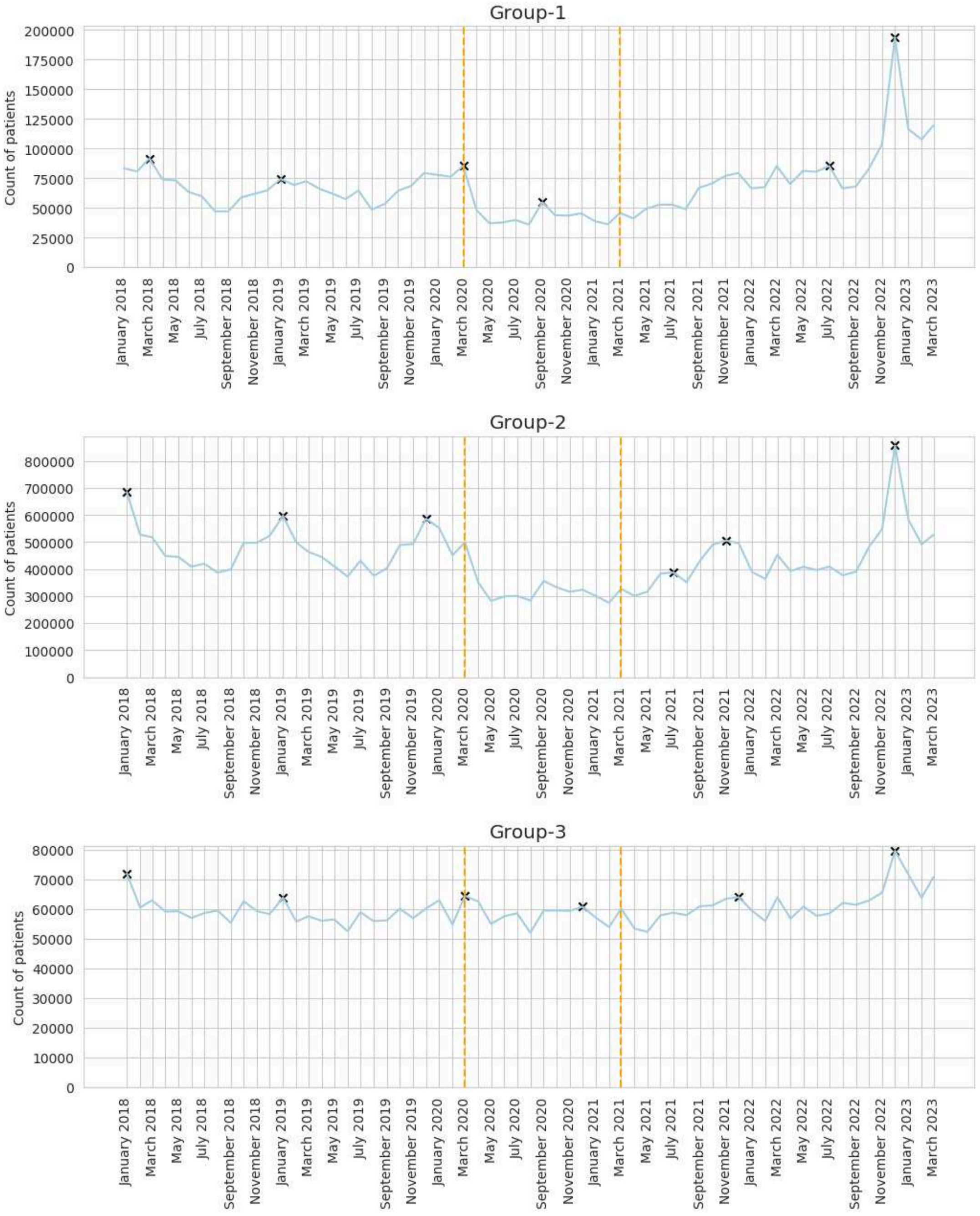

Table S1. Codelists used in this study

Codelist type	Codelist description	Link
Antibiotic	Phenoxymethylpenicillin	https://codelists.opensafely.org/codelist/opensafely/phenoxymethylpenicillin-oral-preparations-only/14b427f8/
	Amoxicillin	https://codelists.opensafely.org/codelist/opensafely/amoxicillin-oral/164af813/
	Clarithromycin	https://codelists.opensafely.org/codelist/opensafely/clarithromycin-oral/080684b6/
	Erythromycin	https://codelists.opensafely.org/codelist/opensafely/erythromycin-oral/7d3b84dd/
	Azithromycin	https://codelists.opensafely.org/codelist/opensafely/azithromycin-oral/42faf594/
	Flucloxacillin	https://codelists.opensafely.org/codelist/opensafely/flucloxacillin-oral/35dbdb0f/
	Cefalexin	https://codelists.opensafely.org/codelist/opensafely/cefalexin-oral/4eeb4e44/
	Co-amoxiclav	https://codelists.opensafely.org/codelist/opensafely/co-amoxiclav-oral/67fac179/
Clinical event	Scarlet Fever	https://opencodelists.org/codelist/opensafely/scarlet-fever/1ba70f02/
	Sore throat/tonsillitis	https://opencodelists.org/codelist/opensafely/group-a-streptococcal-sore-throat/2924ced0/
	Invasive Strep A	https://.opencodelists.org/codelist/opensafely/invasive-group-a-strep/42344205/

Table S2: The max and min count and rate per 1,000 patients for scarlet fever, sore throat/tonsillitis, or iGAS in each season (September through August). Counts have been rounded to the nearest 10 and the rate computed with the rounded number.

	gas_year	2017/18		2018/19		2019/20		2020/21		2021/22		2022/23		2022/23 v 2017/18
		Count	Rate (95% CI)	Count	Rate (95% CI)	Count	Rate (95% CI)	Count	Rate (95% CI)	Count	Rate (95% CI)	Count	Rate (95% CI)	Rate Ratio (95% CI)
name	type													
Invasive Strep A	max	30	0.00 (0.00 to 0.00)	20	0.00 (0.00 to 0.00)	20	0.00 (0.00 to 0.00)	10	0.00 (0.00 to 0.00)	40	0.00 (0.00 to 0.00)	140	0.01 (0.00 to 0.01)	4.37 (2.94 to 6.48)
	min	10	0.00 (0.00 to 0.00)	10	0.00 (0.00 to 0.00)	10	0.00 (0.00 to 0.00)	10	0.00 (0.00 to 0.00)	10	0.00 (0.00 to 0.00)	20	0.00 (0.00 to 0.00)	1.89 (0.89 to 4.05)
Scarlet Fever	max	4570	0.19 (0.19 to 0.20)	1240	0.05 (0.05 to 0.05)	1600	0.06 (0.06 to 0.07)	150	0.01 (0.01 to 0.01)	1720	0.07 (0.06 to 0.07)	13040	0.51 (0.50 to 0.52)	2.68 (2.59 to 2.77)
	min	320	0.01 (0.01 to 0.01)	260	0.01 (0.01 to 0.01)	50	0.00 (0.00 to 0.00)	70	0.00 (0.00 to 0.00)	170	0.01 (0.01 to 0.01)	610	0.02 (0.02 to 0.03)	1.81 (1.58 to 2.07)
Sore Throat Tonsillitis	max	91510	3.84 (3.82 to 3.87)	75070	3.10 (3.07 to 3.12)	77470	3.15 (3.13 to 3.17)	30860	1.25 (1.23 to 1.26)	58630	2.31 (2.30 to 2.33)	135860	5.33 (5.30 to 5.36)	1.39 (1.38 to 1.40)
	min	40130	1.67 (1.65 to 1.68)	41000	1.68 (1.66 to 1.69)	16170	0.65 (0.64 to 0.66)	15940	0.64 (0.63 to 0.65)	41210	1.63 (1.62 to 1.65)	43880	1.73 (1.71 to 1.74)	1.04 (1.02 to 1.05)
Any GAS Clinical Event	max	93,340	3.91 (3.88 to 3.93)	75,930	3.13 (3.11 to 3.15)	78,830	3.20 (3.18 to 3.23)	30,940	1.25 (1.24 to 1.27)	60,040	2.37 (2.35 to 2.39)	146,260	5.74 (5.71 to 5.77)	1.47 (1.46 to 1.48)
	min	40,420	1.68 (1.66 to 1.70)	41,230	1.69 (1.67 to 1.70)	16,240	0.66 (0.65 to 0.67)	16,000	0.64 (0.63 to 0.65)	41,520	1.65 (1.63 to 1.66)	44,430	1.75 (1.73 to 1.77)	1.04 (1.03 to 1.06)

Table S3. The percent of prescriptions that had a GAS indication up to 14 days prior or 7 days after the prescribing event and the percent of clinical events that had a prescription up to 7 days prior and 14 days after the clinical event at the end of the study period (March 2023). Those with Invasive Strep A (iGAS) may be more likely to be treated in secondary care.

		**Percent (95% CI)**
**Medication with clinical any**	Phenoxymethylpenicillin	40.6 (40.3 to 40.9)
	Clarithromycin	11.3 (11.0 to 11.5)
	Erythromycin	10.0 (9.5 to 10.4)
	Amoxicillin	3.2 (3.1 to 3.2)
	Co_Amoxiclav	1.3 (1.2 to 1.4)
	Cefalexin	0.9 (0.8 to 1.0)
	Azithromycin	0.7 (0.6 to 0.8)
	Flucloxacillin	0.3 (0.2 to 0.3)
**Clinical with medication any**	Scarlet_Fever	89.6 (88.6 to 90.6)
	Sore_Throat_Tonsillitis	74.7 (74.4 to 75.0)
	Invasive_Strep_A	28.6 (18.0 to 39.2)

Table S4. The max and min count and rate per 1,000 patients with an antibiotic prescription and a record of scarlet fever, sore throat/tonsillitis, or iGAS up to 14 days prior or 7 days after the prescribing event for each season (September through August). Antibiotics are grouped as phenoxymethylpenicillin (Group 1) as the first line antibiotic for GAS infection, macrolides, amoxicillin and flucloxacillin (Group 2) as the antibiotics recommended in case of non-availability or penicillin allergy, and cefalexin and co-amoxiclav (Group 3) as reserved broad-spectrum. Counts have been rounded to the nearest 10 and the rate computed with the rounded number.

	gas_year	2017/18		2018/19		2019/20		2020/21		2021/22		2022/23		2022/23 v 2017/18
		Count	Rate (95% CI)	Count	Rate (95% CI)	Count	Rate (95% CI)	Count	Rate (95% CI)	Count	Rate (95% CI)	Count	Rate (95% CI)	Rate Ratio (95% CI)
name	type													
Group-1	max	48990	2.05 (2.03 to 2.07)	35680	1.47 (1.46 to 1.49)	37910	1.54 (1.53 to 1.56)	15610	0.63 (0.62 to 0.64)	33560	1.32 (1.31 to 1.34)	71440	2.80 (2.78 to 2.82)	1.37 (1.35 to 1.38)
	min	20510	0.85 (0.84 to 0.86)	20830	0.85 (0.84 to 0.86)	7950	0.32 (0.31 to 0.33)	8140	0.33 (0.32 to 0.33)	21280	0.85 (0.84 to 0.86)	24290	0.96 (0.94 to 0.97)	1.12 (1.10 to 1.14)
Group-2	max	21000	0.88 (0.87 to 0.89)	16790	0.69 (0.68 to 0.70)	17560	0.71 (0.70 to 0.72)	6300	0.25 (0.25 to 0.26)	11950	0.47 (0.46 to 0.48)	51690	2.03 (2.01 to 2.04)	2.30 (2.26 to 2.34)
	min	7990	0.33 (0.32 to 0.34)	7860	0.32 (0.31 to 0.33)	3010	0.12 (0.12 to 0.13)	3060	0.12 (0.12 to 0.13)	8120	0.32 (0.32 to 0.33)	8590	0.34 (0.33 to 0.35)	1.02 (0.99 to 1.05)
Group-3	max	930	0.04 (0.04 to 0.04)	650	0.03 (0.02 to 0.03)	690	0.03 (0.03 to 0.03)	280	0.01 (0.01 to 0.01)	480	0.02 (0.02 to 0.02)	2410	0.09 (0.09 to 0.10)	2.42 (2.24 to 2.61)
	min	440	0.02 (0.02 to 0.02)	390	0.02 (0.01 to 0.02)	180	0.01 (0.01 to 0.01)	180	0.01 (0.01 to 0.01)	320	0.01 (0.01 to 0.01)	310	0.01 (0.01 to 0.01)	0.67 (0.58 to 0.77)

Table S5. The max and min count and rate per 1,000 patients with an antibiotic prescription and a record of scarlet fever, sore throat/tonsillitis, or iGAS up to 14 days prior or 7 days after the prescribing event for each season (September through August). Counts have been rounded to the nearest 10 and the rate computed with the rounded number.

	gas_year	2017/18		2018/19		2019/20		2020/21		2021/22		2022/23		2022/23 v 2017/18
		Count	Rate (95% CI)	Count	Rate (95% CI)	Count	Rate (95% CI)	Count	Rate (95% CI)	Count	Rate (95% CI)	Count	Rate (95% CI)	Rate Ratio (95% CI)
name	type													
Amoxicillin	max	9880	0.41 (0.41 to 0.42)	7940	0.33 (0.32 to 0.33)	8440	0.34 (0.34 to 0.35)	2660	0.11 (0.10 to 0.11)	4630	0.18 (0.18 to 0.19)	26770	1.05 (1.04 to 1.06)	2.53 (2.47 to 2.59)
	min	3120	0.13 (0.13 to 0.13)	3020	0.12 (0.12 to 0.13)	920	0.04 (0.03 to 0.04)	1030	0.04 (0.04 to 0.04)	2820	0.11 (0.11 to 0.12)	3370	0.13 (0.13 to 0.14)	1.02 (0.98 to 1.08)
Azithromycin	max	150	0.01 (0.01 to 0.01)	130	0.01 (0.00 to 0.01)	120	0.00 (0.00 to 0.01)	50	0.00 (0.00 to 0.00)	100	0.00 (0.00 to 0.00)	1180	0.05 (0.04 to 0.05)	7.37 (6.22 to 8.74)
	min	60	0.00 (0.00 to 0.00)	60	0.00 (0.00 to 0.00)	30	0.00 (0.00 to 0.00)	20	0.00 (0.00 to 0.00)	40	0.00 (0.00 to 0.00)	80	0.00 (0.00 to 0.00)	1.26 (0.90 to 1.77)
Cefalexin	max	270	0.01 (0.01 to 0.01)	190	0.01 (0.01 to 0.01)	210	0.01 (0.01 to 0.01)	90	0.00 (0.00 to 0.00)	160	0.01 (0.01 to 0.01)	1100	0.04 (0.04 to 0.05)	3.81 (3.33 to 4.35)
	min	120	0.00 (0.00 to 0.01)	100	0.00 (0.00 to 0.00)	40	0.00 (0.00 to 0.00)	60	0.00 (0.00 to 0.00)	100	0.00 (0.00 to 0.00)	90	0.00 (0.00 to 0.00)	0.71 (0.54 to 0.93)
Clarithromycin	max	7120	0.30 (0.29 to 0.31)	6110	0.25 (0.25 to 0.26)	6450	0.26 (0.26 to 0.27)	2680	0.11 (0.10 to 0.11)	5530	0.22 (0.21 to 0.22)	16460	0.65 (0.64 to 0.66)	2.16 (2.10 to 2.22)
	min	3300	0.14 (0.13 to 0.14)	3410	0.14 (0.14 to 0.15)	1580	0.06 (0.06 to 0.07)	1490	0.06 (0.06 to 0.06)	3730	0.15 (0.14 to 0.15)	4110	0.16 (0.16 to 0.17)	1.18 (1.13 to 1.24)
Co Amoxiclav	max	660	0.03 (0.03 to 0.03)	480	0.02 (0.02 to 0.02)	500	0.02 (0.02 to 0.02)	200	0.01 (0.01 to 0.01)	330	0.01 (0.01 to 0.01)	1310	0.05 (0.05 to 0.05)	1.85 (1.69 to 2.04)
	min	310	0.01 (0.01 to 0.01)	280	0.01 (0.01 to 0.01)	120	0.00 (0.00 to 0.01)	120	0.00 (0.00 to 0.01)	220	0.01 (0.01 to 0.01)	220	0.01 (0.01 to 0.01)	0.67 (0.57 to 0.80)
Erythromycin	max	3690	0.15 (0.15 to 0.16)	2370	0.10 (0.09 to 0.10)	2290	0.09 (0.09 to 0.10)	870	0.03 (0.03 to 0.04)	1540	0.06 (0.06 to 0.06)	6740	0.26 (0.26 to 0.27)	1.71 (1.65 to 1.78)
	min	1280	0.05 (0.05 to 0.06)	1110	0.05 (0.04 to 0.05)	380	0.02 (0.01 to 0.02)	420	0.02 (0.02 to 0.02)	880	0.03 (0.03 to 0.04)	850	0.03 (0.03 to 0.04)	0.63 (0.58 to 0.69)
Flucloxacillin	max	340	0.01 (0.01 to 0.02)	290	0.01 (0.01 to 0.01)	310	0.01 (0.01 to 0.01)	140	0.01 (0.00 to 0.01)	250	0.01 (0.01 to 0.01)	540	0.02 (0.02 to 0.02)	1.49 (1.30 to 1.70)
	min	230	0.01 (0.01 to 0.01)	190	0.01 (0.01 to 0.01)	90	0.00 (0.00 to 0.00)	70	0.00 (0.00 to 0.00)	130	0.01 (0.00 to 0.01)	180	0.01 (0.01 to 0.01)	0.74 (0.61 to 0.90)
Phenoxymethyl penicillin	max	48990	2.05 (2.03 to 2.07)	35680	1.47 (1.46 to 1.49)	37910	1.54 (1.53 to 1.56)	15610	0.63 (0.62 to 0.64)	33560	1.32 (1.31 to 1.34)	71440	2.80 (2.78 to 2.82)	1.37 (1.35 to 1.38)
	min	20510	0.85 (0.84 to 0.86)	20830	0.85 (0.84 to 0.86)	7950	0.32 (0.31 to 0.33)	8140	0.33 (0.32 to 0.33)	21280	0.85 (0.84 to 0.86)	24290	0.96 (0.94 to 0.97)	1.12 (1.10 to 1.14)

Table S6. The max and min count and rate per 1,000 patients with a phenoxymethylpenicillin prescription and a record of scarlet fever, sore throat/tonsillitis, or iGAS up to 14 days prior or 7 days after the prescribing event for each season (September through August), by region. Counts have been rounded to the nearest 10 and the rate computed with the rounded number.

	gas_year	2017/18		2018/19		2019/20		2020/21		2021/22		2022/23		2023 v 2018
		Count	Rate (95% CI)	Count	Rate (95% CI)	Count	Rate (95% CI)	Count	Rate (95% CI)	Count	Rate (95% CI)	Count	Rate (95% CI)	Rate Ratio (95% CI)
group	type													
East	max	12,210	2.21 (2.17 to 2.25)	8,960	1.60 (1.56 to 1.63)	9,530	1.67 (1.64 to 1.71)	3,680	0.63 (0.61 to 0.65)	7,960	1.37 (1.34 to 1.40)	17,650	3.02 (2.98 to 3.07)	1.37 (1.34 to 1.40)
	min	5,030	0.90 (0.88 to 0.93)	5,050	0.90 (0.88 to 0.93)	1,850	0.32 (0.31 to 0.34)	1,820	0.32 (0.30 to 0.33)	4,990	0.86 (0.83 to 0.88)	5,750	0.99 (0.96 to 1.01)	1.10 (1.06 to 1.14)
East Midlands	max	8,840	2.12 (2.08 to 2.17)	6,750	1.59 (1.56 to 1.63)	7,380	1.72 (1.68 to 1.76)	2,980	0.69 (0.67 to 0.72)	6,120	1.39 (1.35 to 1.42)	13,530	3.05 (3.00 to 3.10)	1.44 (1.40 to 1.48)
	min	3,720	0.89 (0.86 to 0.92)	3,760	0.90 (0.87 to 0.92)	1,490	0.35 (0.33 to 0.36)	1,560	0.36 (0.34 to 0.38)	4,120	0.95 (0.92 to 0.98)	4,500	1.02 (0.99 to 1.05)	1.15 (1.10 to 1.20)
London	max	1,940	1.26 (1.20 to 1.31)	1,650	1.04 (0.99 to 1.09)	1,910	1.15 (1.10 to 1.20)	750	0.43 (0.40 to 0.46)	1,540	0.85 (0.81 to 0.90)	2,830	1.55 (1.50 to 1.61)	1.24 (1.17 to 1.31)
	min	900	0.58 (0.54 to 0.61)	1,000	0.61 (0.58 to 0.65)	370	0.22 (0.20 to 0.24)	400	0.24 (0.21 to 0.26)	950	0.53 (0.50 to 0.56)	1,180	0.65 (0.62 to 0.69)	1.13 (1.04 to 1.24)
North East	max	2,450	2.15 (2.07 to 2.24)	1,870	1.63 (1.55 to 1.70)	1,820	1.57 (1.49 to 1.64)	790	0.68 (0.63 to 0.73)	1,630	1.38 (1.32 to 1.45)	3,160	2.66 (2.57 to 2.76)	1.24 (1.17 to 1.31)
	min	1,010	0.88 (0.83 to 0.94)	940	0.81 (0.76 to 0.87)	430	0.37 (0.34 to 0.41)	440	0.38 (0.34 to 0.41)	910	0.78 (0.73 to 0.83)	1,050	0.89 (0.84 to 0.94)	1.01 (0.92 to 1.10)
North West	max	5,370	2.54 (2.47 to 2.61)	3,790	1.77 (1.72 to 1.83)	3,640	1.69 (1.64 to 1.75)	1,780	0.82 (0.79 to 0.86)	3,540	1.61 (1.56 to 1.67)	7,740	3.51 (3.43 to 3.59)	1.38 (1.33 to 1.43)
	min	2,010	0.94 (0.90 to 0.99)	2,040	0.95 (0.91 to 0.99)	850	0.39 (0.37 to 0.42)	930	0.43 (0.40 to 0.46)	2,220	1.02 (0.97 to 1.06)	2,550	1.16 (1.11 to 1.20)	1.23 (1.16 to 1.30)
South East	max	2,970	1.87 (1.81 to 1.94)	2,000	1.25 (1.19 to 1.30)	1,890	1.17 (1.11 to 1.22)	850	0.52 (0.48 to 0.55)	1,720	1.04 (0.99 to 1.09)	3,770	2.28 (2.20 to 2.35)	1.22 (1.16 to 1.27)
	min	1,160	0.73 (0.69 to 0.77)	1,130	0.71 (0.67 to 0.75)	410	0.25 (0.23 to 0.28)	410	0.25 (0.23 to 0.28)	1,050	0.64 (0.60 to 0.68)	1,260	0.76 (0.72 to 0.80)	1.05 (0.97 to 1.14)
South West	max	5,100	1.55 (1.51 to 1.59)	3,470	1.03 (1.00 to 1.07)	3,800	1.11 (1.08 to 1.15)	1,500	0.43 (0.41 to 0.45)	3,460	0.98 (0.95 to 1.02)	7,480	2.12 (2.07 to 2.17)	1.37 (1.32 to 1.42)
	min	2,220	0.67 (0.64 to 0.70)	1,900	0.57 (0.55 to 0.60)	740	0.22 (0.20 to 0.23)	830	0.24 (0.22 to 0.26)	1,990	0.57 (0.55 to 0.60)	2,550	0.72 (0.70 to 0.75)	1.08 (1.02 to 1.15)
West Midlands	max	2,160	2.16 (2.07 to 2.25)	1,760	1.73 (1.65 to 1.81)	1,740	1.70 (1.62 to 1.78)	890	0.87 (0.81 to 0.93)	1,500	1.46 (1.39 to 1.53)	3,120	3.02 (2.92 to 3.13)	1.40 (1.33 to 1.48)
	min	900	0.89 (0.83 to 0.95)	920	0.90 (0.84 to 0.96)	420	0.41 (0.37 to 0.45)	400	0.39 (0.35 to 0.43)	920	0.90 (0.84 to 0.95)	1,130	1.10 (1.03 to 1.16)	1.23 (1.13 to 1.34)
Yorkshire and The Humber	max	7,920	2.25 (2.20 to 2.30)	5,940	1.67 (1.63 to 1.71)	6,590	1.84 (1.79 to 1.88)	2,750	0.76 (0.73 to 0.78)	5,950	1.62 (1.58 to 1.66)	11,820	3.20 (3.14 to 3.26)	1.42 (1.38 to 1.46)
	min	3,540	1.00 (0.97 to 1.03)	3,560	1.00 (0.96 to 1.03)	1,370	0.38 (0.36 to 0.40)	1,350	0.37 (0.35 to 0.39)	3,690	1.01 (0.98 to 1.04)	4,240	1.15 (1.12 to 1.19)	1.15 (1.10 to 1.20)

Table S7. The max and min count and rate per 1,000 patients with a phenoxymethylpenicillin prescription and a record of scarlet fever, sore throat/tonsillitis, or iGAS up to 14 days prior or 7 days after the prescribing event for each season (September through August), by age band. Counts have been rounded to the nearest 10 and the rate computed with the rounded number.

	gas_year	2017/18		2018/19		2019/20		2021/21		2021/22		2022/23		2022/23 v 2017/18
		Count	Rate (95% CI)	Count	Rate (95% CI)	Count	Rate (95% CI)	Count	Rate (95% CI)	Count	Rate (95% CI)	Count	Rate (95% CI)	Rate Ratio (95% CI)
group	type													
0-4	max	8530	6.59 (6.45 to 6.73)	6610	5.14 (5.01 to 5.26)	6930	5.44 (5.32 to 5.57)	3310	2.71 (2.62 to 2.80)	5970	4.95 (4.83 to 5.08)	14490	12.05 (11.86 to 12.25)	1.83 (1.78 to 1.88)
	min	2540	1.97 (1.89 to 2.05)	2780	2.16 (2.08 to 2.24)	670	0.53 (0.49 to 0.57)	810	0.66 (0.61 to 0.70)	2490	2.05 (1.97 to 2.13)	3100	2.58 (2.49 to 2.67)	1.31 (1.24 to 1.38)
5-9	max	9490	6.56 (6.43 to 6.69)	5580	3.83 (3.73 to 3.93)	6100	4.18 (4.08 to 4.28)	1900	1.31 (1.25 to 1.37)	6860	4.78 (4.66 to 4.89)	17180	11.97 (11.79 to 12.14)	1.82 (1.78 to 1.87)
	min	1880	1.30 (1.24 to 1.36)	2070	1.42 (1.36 to 1.48)	750	0.52 (0.48 to 0.55)	680	0.47 (0.43 to 0.51)	1880	1.30 (1.24 to 1.36)	3580	2.49 (2.41 to 2.58)	1.92 (1.82 to 2.03)
10-14	max	4940	3.67 (3.57 to 3.78)	3520	2.52 (2.43 to 2.60)	4200	2.93 (2.85 to 3.02)	2830	1.95 (1.87 to 2.02)	3040	2.01 (1.94 to 2.08)	11300	7.36 (7.22 to 7.49)	2.00 (1.94 to 2.07)
	min	1440	1.05 (0.99 to 1.10)	1350	0.95 (0.90 to 1.00)	510	0.35 (0.32 to 0.38)	530	0.36 (0.33 to 0.39)	1760	1.16 (1.10 to 1.21)	2580	1.69 (1.62 to 1.76)	1.61 (1.51 to 1.72)
15-44	max	21780	2.39 (2.36 to 2.42)	17290	1.87 (1.84 to 1.90)	18580	1.97 (1.94 to 2.00)	9010	0.93 (0.91 to 0.95)	15250	1.55 (1.53 to 1.58)	24040	2.42 (2.38 to 2.45)	1.01 (0.99 to 1.03)
	min	12220	1.33 (1.31 to 1.35)	11190	1.22 (1.19 to 1.24)	4550	0.48 (0.47 to 0.49)	4960	0.52 (0.51 to 0.53)	12400	1.27 (1.25 to 1.29)	12950	1.31 (1.29 to 1.33)	0.98 (0.96 to 1.01)
45-64	max	3540	0.57 (0.55 to 0.58)	2880	0.45 (0.44 to 0.47)	3220	0.50 (0.48 to 0.52)	1170	0.18 (0.17 to 0.19)	2310	0.35 (0.34 to 0.37)	3640	0.56 (0.54 to 0.57)	0.98 (0.94 to 1.03)
	min	1880	0.30 (0.29 to 0.31)	1630	0.26 (0.25 to 0.27)	830	0.13 (0.12 to 0.14)	870	0.13 (0.13 to 0.14)	1440	0.22 (0.21 to 0.23)	1630	0.25 (0.24 to 0.26)	0.83 (0.78 to 0.89)
65-74	max	560	0.23 (0.21 to 0.25)	460	0.19 (0.17 to 0.20)	500	0.20 (0.18 to 0.22)	240	0.10 (0.08 to 0.11)	390	0.16 (0.14 to 0.17)	550	0.22 (0.20 to 0.24)	0.97 (0.86 to 1.09)
	min	340	0.14 (0.12 to 0.15)	320	0.13 (0.12 to 0.14)	170	0.07 (0.06 to 0.08)	160	0.06 (0.05 to 0.07)	240	0.10 (0.08 to 0.11)	270	0.11 (0.10 to 0.12)	0.79 (0.67 to 0.92)
75+	max	230	0.12 (0.10 to 0.13)	210	0.10 (0.09 to 0.12)	260	0.12 (0.11 to 0.14)	150	0.07 (0.06 to 0.08)	200	0.09 (0.08 to 0.10)	260	0.11 (0.10 to 0.12)	0.96 (0.80 to 1.14)
	min	160	0.08 (0.07 to 0.09)	140	0.07 (0.06 to 0.08)	120	0.06 (0.05 to 0.07)	100	0.05 (0.04 to 0.06)	130	0.06 (0.05 to 0.07)	180	0.08 (0.07 to 0.09)	0.97 (0.78 to 1.19)

Table S8. Count of items relating to Group A strep treatment dispensed under Serious Shortage Protocol according to Freedom of Information (FOI) requests 30959 (covering Oct 2019 to Oct 2022) and 01234 (covering Nov 2022 to Apr 2023). 4 cefaclor items have been excluded as cefaclor was not included in this analysis. Code can be found https://github.com/opensafely/strepa_scarlet/blob/main/analysis/ssp-analysis/SSP-antibiotics.ipynb

Antibiotic	Items
Amoxicillin	20,320
Phenoxymethylpenicillin	11,875
Flucloxacillin	1,553
Clarithromycin	747
Cefalexin	473
Erythromycin	288
Co-amoxiclav	190
Azithromycin	12
Total	35,458
